# Structural brain MRI abnormalities in *SCN1A*-, *SCN2A*-, *SCN3A*-, and *SCN8A*-related epilepsies: a cohort study

**DOI:** 10.3389/fneur.2025.1706132

**Published:** 2026-01-07

**Authors:** Daewoong Ahn, Daehyun Kim, Hyeon Deok Sang, Heeseung Cho, Ara Ko, Na-Young Shin, Hoon-Chul Kang, Joon Soo Lee, Lip-Yuen Teng, Se Hee Kim

**Affiliations:** 1 Yonsei University College of Medicine, Seoul, Republic of Korea; 2 Division of Pediatric Neurology, Severance Children’s Hospital, Seoul, Republic of Korea; 3 Department of Pediatrics, Yonsei University College of Medicine, Seoul, Republic of Korea; 4 Department of Radiology, Severance Hospital, Yonsei University Health System, Seoul, Republic of Korea; 5 Pediatric Neurology Unit, Department of Pediatrics, Hospital Tuanku Ja’afar, Seremban, Malaysia

**Keywords:** Dravet syndrome, epilepsy, epileptic encephalopathies, genetic, neurodevelopmental disorders, sodium channels, sodium channelopathies

## Abstract

**Purpose:**

To characterize the prevalence and patterns of structural brain magnetic resonance imaging (MRI) abnormalities in children with genetically confirmed *SCN1A*-, *SCN2A*-, *SCN3A*-, or *SCN8A*-related epilepsy and to identify genotype-specific imaging features.

**Methods:**

We retrospectively analyzed brain MRI findings from a single-center cohort of 139 pediatric patients with pathogenic variants of *SCN1A* (*n* = 114), *SCN2A* (*n* = 17), *SCN3A* (*n* = 1), or *SCN8A* (*n* = 7), evaluated between 2010 and 2023. MRI abnormalities were categorized using a standardized classification framework and compared across the genotypes.

**Results:**

MRI abnormalities were identified in 52 of the 139 patients (37.4%). The most common findings were atrophy (21.6%), hippocampal abnormalities (6.5%), white matter signal abnormalities (5.0%) and hypoxic–ischemic encephalopathy (3.6%). Abnormalities were most frequent in the *SCN2A* (70.6%) group, followed by the *SCN8A* (42.9%) and *SCN1A* (31.6%) groups; one patient with *SCN3A*-related epilepsy also exhibited abnormal findings. In *SCN1A*-related epilepsies, the most common abnormalities were cerebral atrophy (15.8%) and hippocampal abnormalities (6.1%). In *SCN2A*-related epilepsies, common abnormalities included atrophy (58.8%), white matter signal abnormalities (17.6%), hypoxic–ischemic encephalopathy (11.8%) and malformations of cortical development (11.8%). In *SCN8A*-related epilepsies, common findings included atrophy (28.6%), hippocampal abnormalities (14.3%), and white matter signal abnormalities (14.3%). One patient with *SCN3A*-related epilepsy exhibited vascular abnormalities.

**Conclusion:**

Contrary to earlier assumptions, structural MRI abnormalities are common in *SCN*-related epilepsies, particularly in *SCN2A*-and *SCN8A*-related epilepsies. MRI may aid in the diagnosis, phenotypic stratification, and prognostication of genetic epilepsy involving voltage-gated sodium channels.

## Introduction

Voltage-gated sodium channelopathies are a group of genetic epilepsies caused by pathogenic variants in the *SCN1A*, *SCN2A*, *SCN3A*, and *SCN8A* genes, which encode the *α*-subunits of neuronal sodium channels critical for action potential generation and neuronal excitability (1). These genes are among the most well-characterized monogenic causes of epilepsy and are increasingly being identified through next-generation sequencing in clinical practice (2). Historically, brain MRI findings in *SCN1A*-related epilepsies, particularly Dravet syndrome, have often been reported to be normal (3). However, emerging evidence indicates that structural abnormalities, such as cerebral or cerebellar atrophy, hippocampal sclerosis, and malformations of cortical development may occur more frequently than previously assumed (4, 5). Abnormal MRI findings have also been reported in *SCN2A*, *SCN3A*, and *SCN8A*-related epilepsies, although most studies to date have been limited to case reports or small series, and imaging patterns remain poorly defined across genotypes (6–8).

Recent studies have demonstrated that pathogenic variants in sodium channel genes, particularly *SCN2A* and *SCN3A*, may be associated not only with epileptic encephalopathies but also with structural brain malformations such as polymicrogyria, focal cortical dysplasia, and other neuronal migration disorders (7, 9–13). Several reports have described *SCN2A*-related epileptic encephalopathy with severe cortical dysplasia and opercular malformation detected on MRI, as well as *SCN3A* variants linked to extensive cortical folding abnormalities (7, 9–13). These observations suggest that there may be possible associations between sodium channel dysfunction and cortical organization and highlight the need for systematic evaluation of MRI features across *SCN*-related epilepsies.

To address this gap, we analyzed structural brain MRI findings in a large single-center pediatric cohort with genetically confirmed *SCN1A*-, *SCN2A*-, *SCN3A*-, and *SCN8A*-related epilepsy. Using consistent radiological classification criteria, we aimed to characterize the prevalence and distribution of imaging abnormalities across genotypes, identify potential genotype-specific imaging patterns, and improve our understanding of the neurobiological underpinnings of sodium channelopathies. We hypothesized that *SCN*-related epilepsies may exhibit gene-specific patterns of MRI abnormalities that could offer diagnostic or prognostic value.

## Methods

### Study design and patient selection

This was a single-center, retrospective cohort study of patients diagnosed with *SCN1A*-, *SCN2A*-, *SCN3A*-, or *SCN8A*-related epilepsy between January 1, 2010, and December 31, 2023, at Severance Children’s Hospital, Seoul, South Korea.

Eligible participants were identified using the in-house database. Additional cases were included if pathogenic or likely pathogenic variants of *SCN1A*, *SCN2A*, *SCN3A*, or *SCN8A* were identified via external genetic testing, including whole-exome sequencing, chromosomal microarray analysis, and Sanger sequencing (14). Only patients clinically diagnosed with epilepsy were included in this study.

Exclusion criteria included the following: (1) absence of MRI scans or descriptive radiology reports; (2) MRI studies limited to functional MRI, magnetic resonance angiography, or magnetic resonance spectroscopy; and (3) patients with postoperative MRI scans only.

### Variant interpretation and classification

Variants were classified following the American College of Medical Genetics and Genomics (ACMG) and Association for Molecular Pathology (AMP) guidelines. The assessment incorporated population frequency data (gnomAD), computational prediction tools such as SIFT and PolyPhen-2, and previously published reports or ClinVar annotations when available. Each variant was categorized as pathogenic, likely pathogenic, or variant of uncertain significance (VUS) (15). Information on the specific nucleotide/protein change, variant type, and ACMG classification was extracted whenever these details were available from clinical records or published sources. When incomplete, variant data were recorded as not available. Parental testing was not routinely performed; thus, inheritance status was noted as unknown unless reported. No functional studies were available for the present cohort.

### MRI acquisition and analysis

MRI scans were obtained using 1.5 T or 3 T scanners with protocols that included axial, coronal, and/or oblique coronal T2-weighted and fluid-attenuated inversion recovery (FLAIR) sequences. Scans performed solely for functional MRI, magnetic resonance angiography, magnetic resonance spectroscopy, or postoperative evaluation were excluded.

All studies were independently reviewed by two experienced pediatric neuroradiologists, and the final interpretations were determined by consensus.

### Classification of radiological findings

The MRI findings were categorized as normal or abnormal. Abnormalities were further classified into the following major groups: (1) atrophy, (2) cerebellar abnormalities, (3) hippocampal abnormalities, (4) hypoxic–ischemic encephalopathy (HIE), (5) malformations of cortical development (MCD), (6) vascular abnormalities, (7) white-matter signal abnormalities, and (8) other structural findings that did not fit the above categories. Atrophy referred to cerebral atrophy, encompassing cortical and/or white-matter volume loss, whereas cerebellar atrophy was analyzed separately under cerebellar abnormalities. Hippocampal abnormalities included hippocampal malformation, volume loss, signal change, or sclerosis. For cases labelled as “HIE,” classification was based on both imaging pattern and perinatal clinical data; if perinatal hypoxia was uncertain or the imaging suggested postictal hypoxic injury, the lesion was designated as “HIE-like.”

Structural MRI findings were also classified according to their presumed pathophysiological mechanism. Primary (developmental) abnormalities were defined as malformations of cortical development plausibly related to the underlying *SCN* gene variant, including polymicrogyria, focal cortical dysplasia, and other neuronal migration disorders. Secondary (acquired or consequential) abnormalities were defined as structural or signal changes likely resulting from epileptic activity, metabolic stress, or hypoxic injury, including cerebral atrophy, hippocampal anomalies, white-matter signal changes, cerebellar atrophy, and HIE-like lesions.

In patients who underwent serial MRI scans, each abnormality was recorded only once; if a particular abnormality was identified on any available scan, the patient was considered positive for that finding.

### Representative cases

Seven illustrative cases were selected to highlight the characteristic MRI features associated with each subtype of sodium channelopathy (*SCN1A*, *SCN2A*, *SCN3A*, and *SCN8A*). Clinical data extracted from the electronic medical records included age at seizure onset, seizure type and duration, frequency and duration of status epilepticus, antiseizure medications administered, and neurodevelopmental outcomes. The neuroimaging data were reviewed using the hospital’s Picture Archiving and Communication System. Status epilepticus was defined as continuous or recurrent seizures lasting 30 min or more without full recovery between episodes.

### Ethical considerations

This study was approved by the Institutional Review Board of Severance Children’s Hospital (IRB No. 4-2024-0742). Informed consent for genetic testing was obtained from all the patients and/or their legal guardians. The requirement for additional informed consent for this retrospective analysis was waived.

## Results

### Patient characteristics and neuroimaging abnormalities

Among 161 eligible participants, 22 were excluded due to missing MRI data, absent descriptive radiology reports, or limited imaging (e.g., magnetic resonance spectroscopy only or incomplete sequences such as missing axial or coronal T2-weighted images). Thus, 139 patients were included in the final analysis (75 males, 64 females) with a median age of 11.4 years (range = 3.9–38.7 years). In this cohort, 111 patients had Dravet syndrome (79.9%), 10 had developmental and epileptic encephalopathy (DEE; 7.2%), 7 had DEE that evolved into Lennox–Gastaut syndrome (LGS; 5.0%), 2 had early-infantile DEE (EIDEE; 1.4%), 2 had infantile epileptic spasms syndrome (IESS; 1.4%), and 3 had genetic epilepsy with febrile seizures plus (GEFS+; 2.2%), while other epilepsy syndromes are detailed in the Supplementary Tables S1, S2. The median age at seizure onset was 5 months (range = 0–48 months), and genetic diagnosis was established at a median age of 2.2 years (range = 0.2–30.8 years). Of these, 114, 17, 1, and 7 patients had SCN1A-, SCN2A-, SCN3A-, and SCN8A-related epilepsy, respectively. In total, 234 MRI scans with adequate documentation were reviewed, with each patient undergoing 1 to 5 MRI studies (median = 1). The median age at initial MRI was 13 months for SCN1A, 6 months for SCN2A, 20 months for SCN3A, and 8 months for SCN8A, reflecting the early-onset nature of seizures across these groups (Table 1).

**Table 1:** Cohort demographics and MRI timing by sodium channelopathy subtype.

Characteristics	*N* (%)	*SCN1A* group	*SCN2A* group	*SCN3A* group	*SCN8A* group
Total *N* of patients^a^	139	114	17	1	7
Male:female	75:64	61:53	13:4	1:0	0:7
Total *N* of MRIs	234	190	31	4	9
Total *N* of MRIs per patient, *n* (%)					
1	80 (57.6)	67 (58.8)	8 (47.1)	0	5 (71.4)
2	35 (25.2)	29 (25.4)	4 (23.5)	0	2 (28.6)
3	15 (10.8)	10 (8.8)	5 (29.4)	0	0
4	6 (4.3)	5 (4.4)	0	1 (100.0)	0
5	3 (2.2)	3 (2.6)	0	0	0
Median age at initial MRI (months, range)^b^	12 (0–350)	13 (1–350)	6 (0–180)	20 (20–20)	8 (0–46)
Distribution of age at initial MRI, *n* (%)^b^					
0 to <12 months	70 (50.4)	50 (43.9)	13 (76.5)	0	7 (100.0)
12 to <24 months	22 (15.8)	19 (16.7)	2 (11.8)	1 (100.0)	0
24 to <48 months	16 (11.5)	14 (12.3)	2 (11.8)	0	0
≥48 months	31 (22.3)	31 (27.2)	0	0	0
Median age at MRI (all scans, months, range)^c^	26 (0–350)	31.5 (1–350)	11 (0–180)	24.5 (20–47)	8 (0–52)
All MRI scans by age group, *n* (%)^c^					
0 to <12 months	80 (34.2)	58 (30.5)	16 (51.6)	0	6 (66.7)
12 to <24 months	32 (13.7)	23 (12.1)	7 (22.6)	2 (50.0)	0
24 to <48 months	34 (14.5)	27 (14.2)	3 (9.7)	2 (50.0)	2 (22.2)
48 months and older	88 (37.6)	82 (43.2)	5 (16.1)	0	1 (11.1)

a Patients with missing MRI descriptions included for completeness of demographic data.

b One initial MRI per patient was used for this analysis.

c Multiple MRIs per patient included in this analysis.

**Figure 1:**
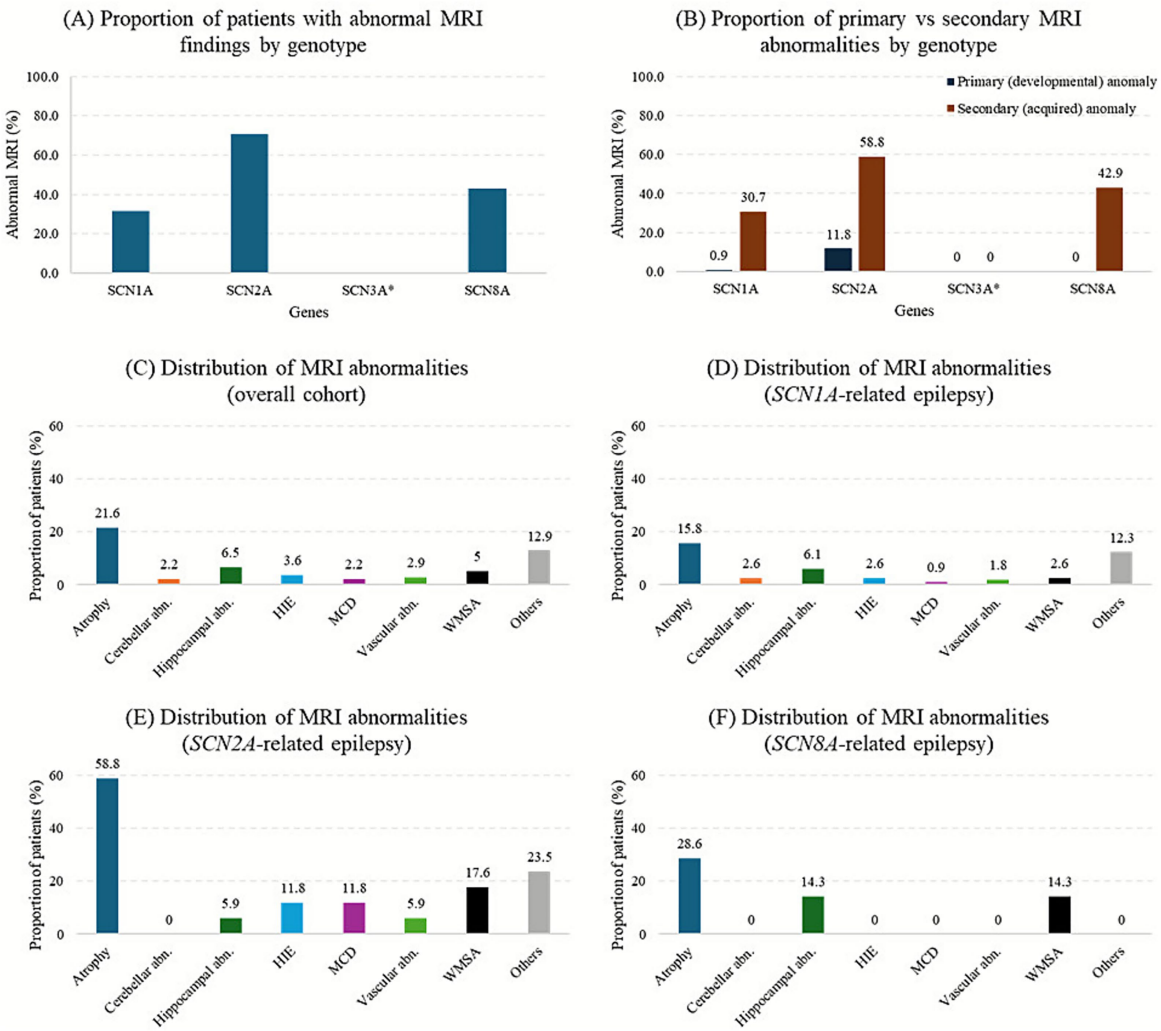
**(A)** Number and percentage of patients with abnormal MRI findings by genetic group. Values in parentheses indicate the percentage of patients with abnormal findings, rounded to one decimal place (100 shown as 100, zero omitted). **(B)** Percentage of patients with primary (developmental) and secondary (acquired) structural abnormalities across *SCN* gene groups. **(C)** Number and percentage of specific abnormal MRI findings in the total sample of patients. **(D–F)** Number and percentage of specific abnormal MRI findings in patients with *SCN1A*, *SCN2A*, and *SCN8A* mutations. “Others” included hydrocephalus, extra-axial calcifications, pineal cysts, arachnoid cysts (e.g., in the middle cranial fossa or quadrigeminal cistern), mild amygdala enlargement, pituitary lesions, thinning of the corpus callosum, and herniation. abn., abnormality(ies); HIE, hypoxic–ischemic encephalopathy; MCD, malformations of cortical development; WMSA, white matter signal abnormalities. ^*^The single *SCN3A* case in our cohort demonstrated superficial siderosis (vascular-related abnormality) without cortical malformation. Because this finding was solitary and considered likely incidental or secondary rather than a gene-specific feature, it was excluded from pooled frequency graphs **(A,B)** to avoid misrepresentation. The *SCN3A* case is retained and described in Table 2 and in the Results text.

Overall, structural MRI abnormalities were identified in 52 of 139 patients (37.4%). Among these, three patients (2.2%) demonstrated primary developmental abnormalities consistent with malformations of cortical development (MCD), while 49 patients (35.3%) exhibited secondary or consequential abnormalities. The highest proportions of MRI abnormalities were observed in the *SCN2A* group (12/17, 70.6%) and *SCN8A* group (3/7, 42.9%), followed by the *SCN1A* group (36/114, 31.6%). The single *SCN3A* case (1/1, 100%) showed superficial siderosis without evidence of cortical malformation. As this vascular-related abnormality was likely incidental or secondary rather than gene-related, it was excluded from pooled frequency graphs (Table 2 and Figures 1A, B).

**Table 2:** Brain magnetic resonance imaging abnormalities observed in patients with sodium channelopathy-related epilepsy.

Magnetic resonance imaging (MRI) findings	Total *N* = 139	*SCN1A* group *N* = 114	*SCN2A* group *N* = 17	*SCN3A* group *N* = 1	*SCN8A* group *N* = 7
Patients with abnormal MRI	52 (37.4)	36 (31.6)	12 (70.6)	1 (100.0)	3 (42.9)
Atrophy	31 (22.3)	18 (15.8)	10 (58.8)	1 (100.0)	2 (28.6)
Cerebellar abnormalities	3 (2.2)	3 (2.6)	0 (0.0)	0 (0.0)	0 (0.0)
Hippocampal abnormalities	9 (6.5)	7 (6.1)	1 (5.9)	0 (0.0)	1 (14.3)
Hypoxic–ischemic encephalopathy	5 (3.6)	3 (2.6)	2 (11.8)	0 (0.0)	0 (0.0)
Malformations of cortical development	3 (2.2)	1 (0.9)	2 (11.8)	0 (0.0)	0 (0.0)
Vascular abnormalities	4 (2.9)	2 (1.8)	1 (5.9)	1 (100.0)	0 (0.0)
White matter signal abnormalities	7 (5.0)	3 (2.6)	3 (17.6)	0 (0.0)	1 (14.3)
Others	18 (12.9)	14 (12.3)	4 (23.5)	0 (0.0)	0 (0.0)

Data represent numbers (percentage).

The single *SCN3A* case (superficial siderosis) is included for completeness but was excluded from pooled frequency graphs due to its solitary and likely incidental/secondary nature.

Across the cohort, the most frequent abnormalities were cerebral atrophy (30/139, 21.6%), hippocampal abnormalities (9/139, 6.5%), white-matter signal abnormalities (7/139, 5.0%), and hypoxic–ischemic encephalopathy-like changes (5/139, 3.6%) (Table 2 and Figure 1C). Cerebral atrophy was identified on the first MRI in 11 of 30 patients (36.7%) at a median age of 11 months (range = 6–100), and on subsequent imaging in 19 of 30 patients (63.3%) at a median age of 16 months (range = 6–183). Hippocampal sclerosis was observed on the first MRI in 5 of 9 patients (55.6%) at a median age of 26 months (range = 12–89) and on follow-up MRI in 4 of 9 patients (44.4%) at a median age of 46 months (range = 40–52). Cerebellar abnormalities were detected on the first neuroimaging in 2 patients (median age, 33 months) and on subsequent MRI in 1 patient at 143 months. MCD was detected in patients with *SCN2A*-related epilepsy (2/17, 11.8%) and *SCN1A*-related epilepsy (1/114, 0.9%).

### *SCN1A*-related epilepsy

In the *SCN1A* group, 36/114 (31.6%) patients exhibited MRI abnormalities. The most common finding was atrophy, observed in 18/114 (15.8%) patients. Hippocampal abnormalities were identified in 7/114 (6.1%) patients, including one with bilateral involvement. Cerebellar abnormalities were observed in 3/114 (2.6%) patients, characterized by cerebellar atrophy, focal tissue loss, and increased T2 signal in the right cerebellar hemisphere. An MCD lesion was noted in 1/114 (0.9%) patients, with a subtle dysplastic cortex in the right frontal lobe suggestive of focal cortical dysplasia (Table 2 and Figure 1D).

**Figure 2:**
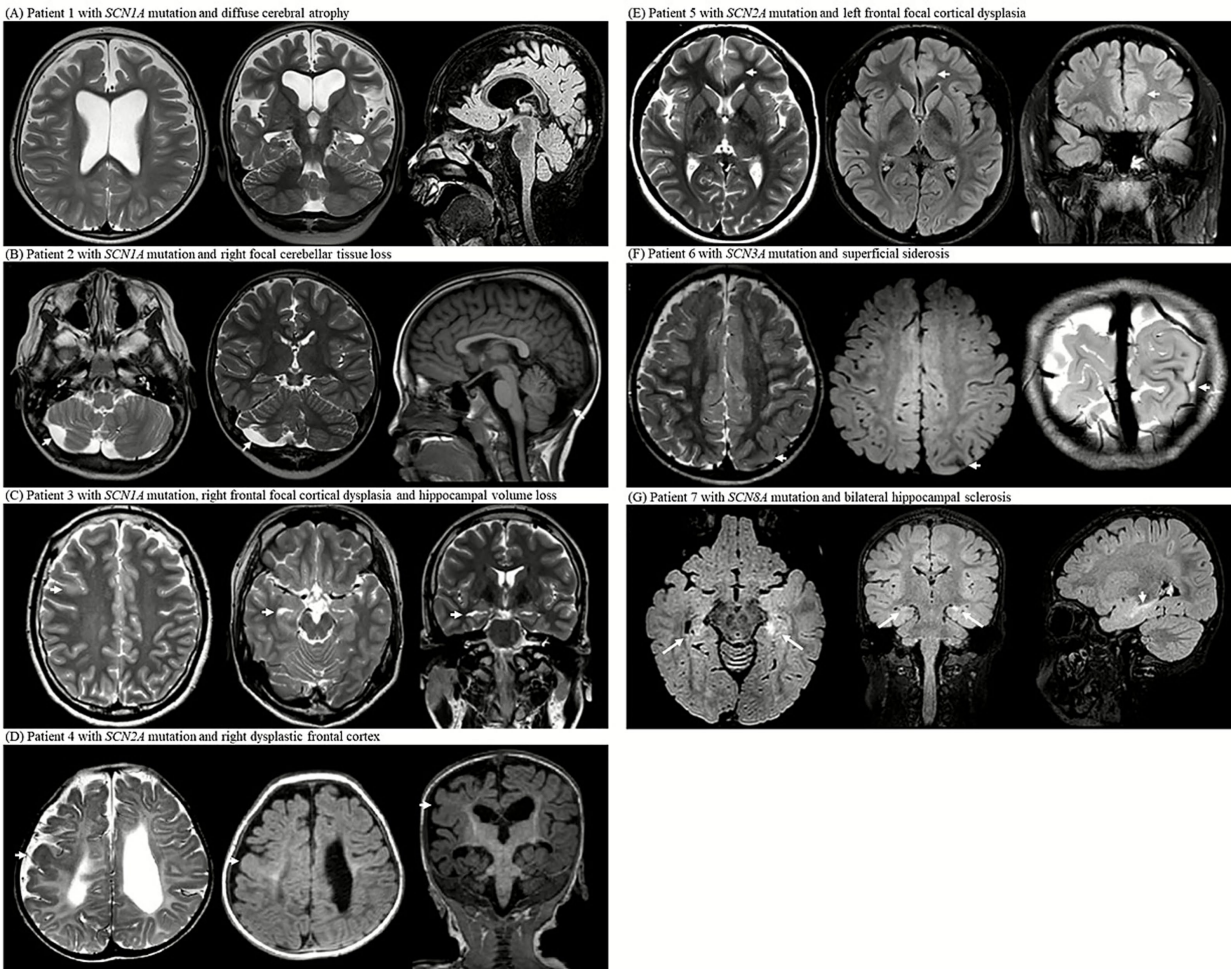
MRI examinations from seven patients with *SCN* gene mutations (*SCN1A*, *SCN2A*, *SCN3A*, and *SCN8A*) reveal a spectrum of structural abnormalities. **(A)** Axial T2-weighted image at the level of the lateral ventricles, coronal T2-weighted image showing the hippocampal formation, and sagittal midline FLAIR image of patient 1 with *SCN1A* mutation; the images demonstrate diffuse cerebral atrophy with marked bilateral ventricular enlargement. **(B)** Axial T2-weighted image at the level of the cerebellum, coronal T2- weighted image through the cerebellar hemispheres, and sagittal midline T1-weighted image of patient 2 with *SCN1A* mutation; the images show focal right cerebellar tissue loss with mild asymmetry. **(C)** Axial T2-weighted image at the centrum semiovale, axial T2-weighted image at the midbrain level, and coronal T2-weighted image showing the hippocampus of patient 3 with *SCN1A* mutation; the images reveal right frontal focal cortical dysplasia and ipsilateral hippocampal volume loss. **(D)** Axial T2-weighted image at the level of the lateral ventricular atrium, axial FLAIR image at the same level, and coronal T1-weighted image of patient 4 with *SCN2A* mutation; the images demonstrate diffuse periventricular leukomalacia with thinning of the corpus callosum and dysplastic cortex of the right frontal lobe. **(E)** Axial T2-weighted image at the level of lateral ventricles, axial FLAIR image at the same level, and coronal FLAIR-weighted image of patient 5 with *SCN2A* mutation; the images show focal cortical thickening and subcortical white matter T2 high signal intensity in left anterior cingulate gyrus, consistent with focal cortical dysplasia. **(F)** Axial T2-weighted image at the centrum semiovale, axial FLAIR image at the same level, and oblique coronal T2-weighted image at the occipital lobe level of patient 6 with *SCN3A* mutation; the images show superficial siderosis along the left parietal convexity. **(G)** Axial FLAIR image at the midbrain level, coronal FLAIR image at the foramen of Monro level, and sagittal midline FLAIR image of patient 7 with *SCN8A* mutation; the images show bilateral hippocampal atrophy with increased T2/FLAIR signal intensity and loss of internal architecture, consistent with hippocampal sclerosis.

*Patient 1*: An 8-year-old boy with a heterozygous *SCN1A* missense mutation (c.680T>G; pIle227Ser) presented with febrile and hemiclonic seizures starting at 4 months of age, including prolonged status epilepticus. Seizures improved significantly (>90% reduction) after cannabidiol was introduced at the age of 4 years, alongside a ketogenic diet. MRI at the age of 6 years revealed diffuse cerebral atrophy and bilateral ventriculomegaly. Earlier developmental assessment at the age of 3 years indicated a profound global delay (Figure 2A).

*Patient 2*: A 23-year-old man with an *SCN1A* insertion mutation (c.1875_1876insG; p.Ser626GlufsTer2) had seizure onset at five months. Despite polytherapy with antiseizure medications, seizures persisted. MRI at the age of 6 years revealed right cerebellar tissue loss (Figure 2B).

*Patient 3*: A 21-year-old male with a heterozygous *SCN1A* nonsense mutation (c.1141C>T; p.Gln381Ter) developed focal motor seizures with status epilepticus at 14 months of age. Despite multiple antiseizure medications and a ketogenic diet, the seizures persisted and worsened upon stiripentol discontinuation. MRI at 19 years of age revealed a dysplastic cortex in the right frontal area and right hippocampal volume loss (Figure 2C).

### *SCN2A*-related epilepsy

In the *SCN2A* group, 12/17 (70.6%) patients exhibited MRI abnormalities. Atrophy was observed in 10/17 (58.8%) patients. White matter signal abnormalities were present in 3/17 (17.6%) patients, including periventricular leukomalacia and diffuse white matter volume loss. Malformations of cortical development were present in 2/17 (11.8%) patients. Hypoxic–ischemic encephalopathy was identified in 2/17 (11.8%) patients with perinatal respiratory distress and infantile-onset seizures, characterized by diffuse gray and white matter volume loss and bilateral globus pallidus T2 hyperintensities (Table 2 and Figure 1E).

*Patient 4*: A 6-year-old girl with a heterozygous *SCN2A* missense mutation (c.4499C>T; p.Ala1500Val) had refractory neonatal-onset epilepsy with bilateral clonic seizures beginning 10 min after birth, evolving into daily myoclonic seizures. MRI at 6 months showed diffuse white matter volume loss and corpus callosum thinning with a dysplastic cortex in the right hemisphere. Electroencephalogram (EEG) confirmed the presence of sharp focal waves. Despite extensive treatment with antiseizure medications, seizures remained intractable. She exhibited profound developmental delays, hypotonia with dystonic posturing, and feeding difficulties requiring gastrostomy. By the age of 2 years, the EEG showed diffuse slow spike-and-wave discharges and paroxysmal fast activity, consistent with Lennox–Gastaut syndrome (Figure 2D).

*Patient 5*: A 20-year-old female with a heterozygous *SCN2A* splice-site mutation (c.3400-2A>G) experienced nocturnal hypermotor seizures at the age of 3 years. MRI at the age of 15 years showed focal cortical thickening and subcortical T2 hyperintensity in the left cingulate gyrus, suggestive of focal cortical dysplasia. EEG confirmed the presence of frontal focal sharp waves. The seizures were well controlled with medication, and development remained normal (Figure 2E).

### *SCN3A*-related epilepsy

The single *SCN3A* patient exhibited superficial siderosis in the left parietal lobe, likely a sequela of a prior subarachnoid hemorrhage (Table 2).

*Patient 6*: An 8-year-old boy with a heterozygous *SCN3A* splice-site mutation (c.3393+2T>G) developed infantile epileptic spasms at 13 months of age and daily atonic seizures with regression. Despite treatment with multiple antiseizure medications, the seizures were drug-resistant. MRI at 18 months of age revealed superficial siderosis of the left parietal lobe. Developmental testing at the age of 3 years showed a global delay (Figure 2F).

### *SCN8A*-related epilepsy

In the *SCN8A* group, 3/7 (42.9%) patients had MRI abnormalities. Atrophy was observed in 2/7 (28.6%) patients, white matter signal abnormalities (T2 hyperintensities) in 1/7 (14.3%) patients, and hippocampal abnormalities (sclerosis) in 1/7 (14.3%) (Table 2 and Figure 1F).

*Patient 7*: A 7-year-old girl with a heterozygous *SCN8A* missense mutation (c.2934C>A; p.Ser978Arg) had seizure onset at 2 weeks, followed by febrile status epilepticus at 2 months, and persistent generalized tonic–clonic seizures. Despite polytherapy with antiseizure medications, seizures persisted. She developed dysphagia, hypothyroidism, growth failure, and global developmental delay. MRI at the age of 4 years revealed bilateral hippocampal atrophy with increased signal intensity. EEG at 18 months showed a slow, disorganized background, whereas EEG at the age of 6 years revealed diffuse fast activity and intermittent sharp waves, predominantly over the frontal regions (Figure 2G).

## Discussion

In this single-center cohort of 139 pediatric patients with genetically confirmed *SCN1A*-, *SCN2A*-, *SCN3A*-, and *SCN8A*-related epilepsies, structural brain MRI abnormalities were identified in 37.4% of the patients. The most frequent finding was atrophy, followed by hippocampal abnormalities, white matter signal abnormalities, and hypoxic–ischemic encephalopathy. These findings challenge the conventional notion that sodium channelopathies, particularly *SCN1A*-related epilepsy, are radiologically silent and underscore the evolving understanding of genotype-specific neuroimaging profiles.

Clinicians should interpret these findings cautiously, considering them in the context of each patient’s clinical and developmental profile. Many of the structural MRI abnormalities observed may be incidental or secondary rather than directly caused by the underlying SCN gene defect, and the considerable variability in MRI findings, even among patients carrying the same gene variant, precludes definitive genotype–phenotype correlations. Nevertheless, the relatively high prevalence of structural abnormalities in this cohort is noteworthy. These observations contribute to a more comprehensive characterization of the neuroimaging spectrum in SCN-related epilepsies and may inform future prospective, longitudinal studies aimed at distinguishing primary disease-related changes from secondary or incidental findings.

### *SCN1A*-related epilepsy

MRI in *SCN1A*-related epilepsies, including Dravet syndrome, has historically been reported as normal, especially in early life. According to International League Against Epilepsy guidance, brain MRI is not required for the diagnosis of Dravet syndrome but is recommended to exclude an alternative structural etiology; identification of a causal structural lesion on MRI constitutes an exclusion criterion (3). However, recently, the increasing recognition of structural abnormalities on follow-up imaging, particularly atrophy and hippocampal sclerosis, has emerged with advances in imaging resolution and wider adoption of longitudinal follow-up protocols (4). Prior studies have reported MRI abnormalities in 11% of *SCN1A*-positive Dravet cohorts and approximately 22% in unselected Dravet cohorts (16, 17). Another study reported that in patients imaged after the age of 3 years, temporal lobe abnormalities, including hippocampal sclerosis or loss of gray-white matter definition, were present in 39% (18). In our cohort, 31.6% of patients exhibited abnormalities, including cerebral atrophy (15.8%) and hippocampal abnormalities (6.1%), which is a proportion that falls between rates reported in mutation-positive cohorts and in patients imaged after age 3 years (16–18). These findings support the accumulated evidence indicating that MRI abnormalities may emerge over time, particularly in patients with prolonged or recurrent seizures (16). The slightly higher prevalence observed in our cohort may reflect the inclusion of older patients, the greater availability of follow-up imaging, and potential genetic homogeneity.

While rare, focal lesions such as cortical dysplasia were observed in our cohort. As previously reported (19), such findings raise diagnostic challenges; the presence of a focal abnormality should not preclude the consideration of an underlying genetic cause such as *SCN1A*. Importantly, this distinction has therapeutic implications, as sodium channel blockers, which are usually the treatment of choice for focal seizures, may worsen seizures in *SCN1A*-related epilepsy.

### *SCN2A*-related epilepsy

The neuroimaging spectrum of *SCN2A*-related epilepsy is notably heterogeneous and appears to correlate with clinical severity. In our cohort, 70.6% of patients demonstrated MRI abnormalities, a rate substantially higher than approximately 40% reported in a prior pediatric *SCN2A* cohort (11). The most frequent abnormality was atrophy (58.8%), which is often observed in patients with early-onset epileptic encephalopathy and severe developmental delay. Two patients with neonatal seizures and perinatal respiratory compromise exhibited imaging patterns consistent with hypoxic–ischemic encephalopathy, raising questions as to whether the observed changes were of genetic origin or reflected perinatal injury. However, the presence of white matter signal abnormalities and malformations of cortical development in other *SCN2A* cases suggests an intrinsic neurodevelopmental contribution. Recently, we reported a genetic diagnosis of neonatal encephalopathy with hypoxic brain damage using targeted gene panel sequencing (20).

Our representative cases further illustrate this clinical-radiological variability: one with profound encephalopathy and diffuse periventricular leukomalacia, and another with later-onset focal epilepsy and focal cortical dysplasia. These data underscore the need for careful phenotypic characterization of neonatal- and infantile-onset epilepsy, particularly when neuroimaging abnormalities do not correlate with the usual disease course of patients with perinatal hypoxic–ischemic encephalopathy.

### *SCN3A*-related epilepsy

*SCN3A*-related epilepsies are increasingly recognized for their strong association with malformations of cortical development, particularly polymicrogyria (7). In contrast to these typical features, our patient demonstrated only superficial siderosis, likely a sequela of a prior subarachnoid hemorrhage without overt cortical malformations. This differs from earlier findings by Zaman et al. (7), who reported that over 75% of the patients had polymicrogyria or other cortical anomalies. Despite the absence of visible malformations on conventional MRI, the patient’s early-onset drug-resistant epilepsy and developmental delay suggested the possibility of subtle or microscopic cortical anomalies that are not detectable on routine imaging. This case highlights the limitations of standard MRI protocols and underscores the potential role of advanced neuroimaging techniques (e.g., 3 T MRI with optimized cortical sequences or diffusion-based mapping) in uncovering hidden structural abnormalities. This also reinforces the importance of individualized imaging approaches, even in single cases in which clinical severity strongly suggests a developmental origin.

### *SCN8A*-related epilepsy

*SCN8A*-related epilepsy is associated with a progressive clinical course and structural changes in the brain. In our cohort, 42.9% of patients demonstrated MRI abnormalities, including cerebral atrophy, white matter signal abnormalities (T2 hyperintensities), and hippocampal abnormalities (sclerosis). These observations are consistent with prior literature: a review of *SCN8A*-related developmental and epileptic encephalopathy reported that brain MRI is normal in approximately half of patients and that, when abnormal, the most common finding is mild diffuse cerebral atrophy (21), and a small single-center cohort documented MRI abnormalities in 28.6% of patients (22). Notably, our representative case showed MRI signs suggestive of bilateral hippocampal sclerosis at the age of 6 years, which was reflective of severe seizure-induced injury. This early-onset development of hippocampal abnormalities emphasizes the importance of follow-up MRI in patients with ongoing seizures. Our findings also suggest that *SCN8A*-related encephalopathies may be underrecognized as progressive imaging disorders, particularly when early scans appear normal. Compared with *SCN1A*, *SCN8A* showed a higher proportion of structural abnormalities, particularly atrophy, in our cohort, consistent with prior descriptions of a more severe clinical phenotype and with longitudinal studies demonstrating progressive cortical and subcortical atrophy in *SCN8A* developmental and epileptic encephalopathy (23). These observations underscore the need for longitudinal imaging in patients with suspected *SCN8A* encephalopathy.

### Study strengths and limitations

This study is one of the largest single-center comparative neuroimaging analyses of *SCN1A*-, *SCN2A*-, *SCN3A*-, and *SCN8A*-related epilepsies. A major strength lies in the integration of detailed phenotypic–genotypic correlations with radiological findings, supported by illustrative case vignettes and longitudinal imaging, where available. The inclusion of a genetically confirmed cohort minimizes diagnostic ambiguity and allows gene-specific neuroimaging characterization.

However, this study had several limitations. First, this was a retrospective study conducted at a single tertiary center, which may limit generalizability and introduce referral bias toward more severe or treatment-resistant cases. Second, the MRI protocols were not fully standardized across all patients or time points, potentially affecting the sensitivity of detecting subtle anomalies, particularly malformations of cortical development. Third, quantitative imaging metrics and volumetric analyses were not performed, which may have enhanced the assessment of progressive atrophy. Fourth, the relatively small sample sizes of the *SCN2A*, *SCN3A*, and *SCN8A* subgroups limit statistical comparisons and warrant cautious interpretation. Finally, MRI abnormalities detected on initial scans may not be directly attributable to the underlying gene defect itself, as they could also reflect secondary or timing-related factors such as disease evolution, seizure burden, or postictal changes at the time of imaging. Such variability complicates causal inference and limits the precision of genotypic–phenotypic correlations with radiological findings.

## Conclusion

Structural brain abnormalities appear to be more common in *SCN*-related epilepsies than previously recognized, particularly in *SCN2A* and *SCN8A*, where MRI changes may reflect both developmental disturbances and progressive injury. Even in *SCN1A*-related epilepsy, historically considered radiologically normal, follow-up imaging revealed a notable proportion with atrophy or hippocampal changes. In this study, it remains uncertain whether these abnormalities are incidental findings or directly related to the underlying gene defect. As this was a descriptive retrospective analysis, the observations should be interpreted with caution and verified in future prospective studies that include appropriate control groups. Some patients with initially normal scans developed new findings on serial MRI, suggesting that repeat imaging may be helpful when clinical or seizure features evolve. Overall, these results support continued use of longitudinal MRI surveillance in sodium channelopathies while emphasizing the need for multicentre studies using standardized imaging methods to clarify their significance. Importantly, these findings are descriptive, and structural abnormalities should not be assumed to result directly from *SCN* gene defects. The heterogeneity of MRI patterns underscores the need for careful clinical interpretation and highlights that genotype–phenotype correlations remain preliminary.

## Data Availability

The original contributions presented in the study are included in the article and its Supplementary material. Further inquiries can be directed to the corresponding authors.
