# Supplementary material for: Structural brain MRI abnormalities in SCN1A-, SCN2A-, SCN3A-, and SCN8A-related epilepsies: a cohort study
Source: Front Neurol. 2026 Jan 7;16:1706132. doi: 10.3389/fneur.2025.1706132 (PMC12819171; doi:10.3389/fneur.2025.1706132)
Supplement: Supplementary file 1 [file Table_1.docx]

**Supplementary Material**

Table S1. Patient characteristics and epilepsy syndromes in our cohort.

| **Characteristics** | **Value*** |
| --- | --- |
| Total participants (eligible) | 161 |
| Excluded due to incomplete MRI data | 22 |
| Final cohort analyzed | 139 |
| Sex (male : female) | 75 : 64 |
| Median age at last follow-up (years) | 11.4 (range 3.9-38.7) |
| Median age at seizure onset (months) | 5 (range 0-48) |
| Median age at genetic diagnosis (years) | 2.2 (range 0.2-30.8) |
| SCN gene distribution   - SCN1A - SCN2A - SCN3A - SCN8A | 114 (82.0%)  17 (12.2%)  1 (0.7%)  7 (5.0%) |
| Epilepsy syndrome   - Dravet syndrome - DEE - DEE→LGS - EIDEE - IESS - GEFS+ - SeLFIE - NE - IE - FLE | 111 (79.9%)  10 (7.2%)  7 (5.0%)  2 (1.4%)  2 (1.4%)  3 (2.2%)  1 (0.7%)  1 (0.7%)  1 (0.7%)  1 (0.7%) |

Abbreviations

DEE, developmental and epileptic encephalopathy; DS, Dravet syndrome; EIDEE, early-infantile developmental and epileptic encephalopathy (Ohtahara syndrome); FLE, frontal lobe epilepsy; GEFS+, genetic epilepsy with febrile seizure plus; IE, infantile epilepsy; IESS, infantile epileptic spasms syndrome; IS, infantile spasms; LGS, Lennox-Gastaut syndrome; NE, neonatal epilepsy; SeLFIE, self-limited familial infantile epilepsy; →, evolution of epilepsy syndrome.

*Data are number (%) or median (range), unless otherwise indicated.

Table S2. Genetic and MRI characteristics of individuals with sodium channelopathy-related epilepsies.

| **ID** | **Age (y)** | **Sex** | **Gene** | **Mutation type** | **Variant** | **Pathogenicity** | **Age at genetic test (y)** | **Number of MRIs** | **Age at serial MRIs (m)*** | **MRI Findings (Age at MRI)** |
| --- | --- | --- | --- | --- | --- | --- | --- | --- | --- | --- |
| Pt1 | 38.7 | F | SCN1A | Spl | c.2556+3A>T | LP | 30.8 | 1 | 277 | NL |
| Pt2 | 33.1 | F | SCN1A | Mis | c.1178G>A, p.Arg393His | P | 16 | 1 | 267 | NL |
| Pt3 | 38.5 | M | SCN1A | Spl | c.4306-1G>A | LP | 30.3 | 1 | 198 | NL |
| Pt4 | 28.5 | F | SCN1A | Non | c.2101C>T, p.Arg701Ter | P | 21.4 | 1 | 116 | NL |
| Pt5 | 32.3 | F | SCN1A | Fs | c.4934del, p.Arg1645GlnfsTer5 | P | 8 | 1 | ***350*** | HS, Lt AmyE (350m) |
| Pt6 | 26.3 | M | SCN1A | Mis | c.716C>A, p.Ala239Asp | LP | 18.8 | 1 | ***93*** | HS, CCA (93m) |
| Pt7 | 24.5 | F | SCN1A | Non | c.5428C>T, p.Gln1810Ter | P | 18.2 | 3 | 39, 45, ***61*** | Multifocal subcortical WM-SA (61m) |
| Pt8 | 29.3 | F | SCN1A | Fs | c.407delG, p.Cys136SerfsTer4 | LP | 23.1 | 1 | 285 | NL |
| Pt9 | 30.4 | M | SCN1A | Spl | c.1170+2T>C | LP | 22.8 | 2 | 158, ***183*** | Lt PL CA (183m) |
| Pt10 | 24.0 | M | SCN1A | Non | c.459G>A, p.Trp153Ter | LP | - | 3 | 86, ***143, 149*** | Rt Cb-SA (143m) |
| Pt11 | 23.7 | M | SCN1A | Fs | c.1875_1876insG, p.Ser626GlufsTer2 | LP | - | 2 | ***36, 82*** | Rt Cb-A (from 36m) |
| Pt12 | 21.2 | M | SCN1A | Spl | c.2176+1C>T | LP | - | 5 | 21, 85, ***97, 98, 103*** | CA (97m) |
| Pt13 | 23.6 | F | SCN1A | Non | c.4219C>T, p.Arg1407Ter | P | - | 1 | 45 | NL |
| Pt14 | 20.3 | M | SCN1A | Non | - | - | - | 2 | ***8, 29*** | CA (8m) |
| Pt15 | 21.8 | M | SCN1A | Non | c.1141C>T, p.Gln381Ter | LP | 14.9 | 4 | ***27, 40, 88, 234*** | FCD (from 17m); HS (from 40m); Lt FL extra-axial calcn (234m) |
| Pt16 | 21.7 | F | SCN1A | Non | - | - | - | 3 | 32, 100, ***137*** | CA (137m) |
| Pt17 | 23.7 | F | SCN1A | Non | c.3779G>A, p.Trp1260Ter | LP | 17.9 | 3 | 69, 112, 197 | NL |
| Pt18 | 22.0 | M | SCN1A | Non | c.992T>A, p.Leu331Ter | LP | 14.4 | 1 | 154 | NL |
| Pt19 | 19.6 | M | SCN1A | - | - | - | - | 4 | 20, 31, 91, 152 | NL |
| Pt20 | 19.1 | M | SCN1A | - | - | - | - | 1 | 96 | NL |
| Pt21 | 22.0 | M | SCN1A | - | - | - | - | 3 | ***100, 106, 150*** | HIE (100m); CA, VE (150m) |
| Pt22 | 17.8 | F | SCN1A | Mis | c.5341T>C, p.Tyr1781His | LP | - | 5 | 7, 8, 27, 83, 191 | NL |
| Pt23 | 19.0 | M | SCN1A | - | - | - | - | 1 | 125 | NL |
| Pt24 | 17.7 | F | SCN1A | - | - | - | - | 3 | 6, 7, 121 | NL |
| Pt25 | 16.9 | M | SCN1A | - | - | - | - | 1 | 8 | NL |
| Pt26 | 17.5 | M | SCN1A | Non | c.2237T>G, p.Leu746Ter | LP | - | 2 | 94, 132 | NL |
| Pt27 | 21.8 | F | SCN1A | Spl | - | - | - | 1 | 75 | NL |
| Pt28 | 16.3 | M | SCN1A | Fs | - | - | - | 4 | 5, 6, 11, 113 | NL |
| Pt29 | 17.1 | M | SCN2A | Mis | c.593T>A, p.Val198Asp | LP | 10.7 | 5 | 15, 15, 22, ***52, 105*** | CA, VE (52m) |
| Pt30 | 16.1 | M | SCN1A | Non | c.3952C>T, p.Arg1318Ter | P | 8.3 | 2 | ***100, 112*** | CA, bilateral OL subcortical WM-SA (100m) |
| Pt31 | 20.5 | F | SCN1A | Mis | c.2665G>A, p.Ala889Thr | LP | - | 1 | 69 | NL |
| Pt32 | 15.7 | F | SCN1A | - | - | - | - | 2 | 8, 77 | NL |
| Pt33 | 19.2 | F | SCN1A | Non | c.1837C>T, p.Arg613Ter | P | - | 1 | 54 | NL |
| Pt34 | 15.9 | F | SCN1A | Fs | c.5565dup, p.Met1856HisfsTer5 | LP | - | 2 | 9, 94 | NL |
| Pt35 | 15.1 | F | SCN1A | Mis | c.5348C>T, p.Ala1783Val | LP | - | 3 | 3, 6, 149 | NL |
| Pt36 | 18.6 | M | SCN1A | Mis | c.272T>C, p.Ile91Thr | LP | 10.9 | 2 | 114, 130 | NL |
| Pt37 | 14.6 | M | SCN1A | Del | 2q24.3 deletion | P | - | 3 | 2, ***7, 18*** | FL CA (7m) |
| Pt38 | 14.3 | M | SCN1A | Mis | - | - | - | 1 | 30 | NL |
| Pt39 | 14.3 | M | SCN1A | Mis | - | - | - | 2 | 7, 9 | NL |
| Pt40 | 17.6 | F | SCN1A | Spl | c.2556+3A>T | LP | 9.8 | 1 | 117 | NL |
| Pt41 | 13.8 | F | SCN1A | Spl | c.2415+1G>A | LP | - | 1 | 9 | NL |
| Pt42 | 19.2 | F | SCN1A | Fs | - | - | - | 4 | ***69, 88, 115, 137*** | HS (from 69m); subacute SDH (137m) |
| Pt43 | 16.3 | M | SCN1A | Mis | c.1006T>C, p.Cys336Arg | P | - | 1 | 110 | NL |
| Pt44 | 14.2 | F | SCN1A | Non | c.4933C>T , p.Arg1645Ter | P | - | 1 | ***11*** | Diffuse CA (11m) |
| Pt45 | 14.0 | F | SCN1A | Mis | c.3785C>T, p.Ala1262Val | LP | 6.2 | 1 | 9 | NL |
| Pt46 | 13.8 | F | SCN2A | Mis | c.2932T>C, p.Phe978Leu | LP | 5.8 | 3 | ***8, 17, 45*** | Periventricular WM-SA, CCA (8m); progressive WM-SA & CCA (from 17m) |
| Pt47 | 14.1 | M | SCN1A | - | - | - | - | 1 | 45 | NL |
| Pt48 | 13.4 | M | SCN1A | Del | Exon 20 deletion | P | - | 4 | 4, 26, 86, ***111*** | Diffuse CA (111m) |
| Pt49 | 15.3 | F | SCN1A | Fs | c.3572delG, p.Cys1191PhefsTer17 | LP | - | 1 | 91 | NL |
| Pt50 | 12.3 | F | SCN8A | Mis | c.5630A>G, p.Asn1877Ser | LP | 6.4 | 2 | 6, 46 | NL |
| Pt51 | 11.7 | M | SCN2A | Mis | c.788C>T, p.Ala263Val | P | 3.9 | 5 | 1, 2, ***16, 48, 78*** | Diffuse CA (16m) |
| Pt52 | 12.6 | M | SCN1A | Mis | c.4133A>C, p.Asn1378Thr | LP | - | 1 | 71 | NL |
| Pt53 | 16.8 | F | SCN1A | Non | c.1129C>T, p.Arg377Ter | P | - | 2 | 14, 126 | NL |
| Pt54 | 12.1 | M | SCN1A | Mis | c.1033T>C, p.Cys345Arg | LP | 5.9 | 1 | 4 | NL |
| Pt55 | 12.5 | F | SCN1A | Fs | c.5503_5506delAAAC, p.Lys1835SerfsTer11 | LP | 4.2 | 2 | 5, 19 | NL |
| Pt56 | 14.7 | F | SCN1A | Fs | c.3819dup, p.Cys1274ValfsTer9 | LP | 8.7 | 2 | 6, 55 | NL |
| Pt57 | 11.6 | M | SCN1A | Mis | c.1121C>A, p.Ser374Tyr | LP | - | 1 | 6 | NL |
| Pt58 | 11.2 | F | SCN1A | Non | c.1345G>T, p.Glu449Ter | LP | - | 2 | 6, ***28*** | AC (28m) |
| Pt59 | 11.4 | M | SCN1A | - | - | - | - | 1 | 13 | NL |
| Pt60 | 11.3 | F | SCN1A | Non | c.1624C>T, p.Arg542Ter | P | - | 1 | 8 | NL |
| Pt61 | 11.8 | M | SCN1A | - | - | - | - | 1 | 35 | NL |
| Pt62 | 15.6 | M | SCN1A | Fs | c.29delG, p.Gly10AspfsTer82 | LP | 7.8 | 2 | ***64, 94*** | FTL CA (from 64) |
| Pt63 | 10.9 | M | SCN1A | Fs | c.1209dup, p.Val404CysfsTer46 | LP | - | 1 | 71 | NL |
| Pt64 | 11.1 | F | SCN1A | Non | c.3733C>T, p.Arg1245Ter | P | - | 1 | 19 | NL |
| Pt65 | 11.0 | M | SCN1A | Mis | c.4317G>C, p.Gln1439His | LP | 3.3 | 2 | 20, ***74*** | Diffuse CA (74m) |
| Pt66 | 10.8 | F | SCN1A | Mis | - | - | - | 2 | 7, 87 | NL |
| Pt67 | 10.8 | F | SCN1A | Non | - | - | - | 1 | 13 | NL |
| Pt68 | 11.9 | M | SCN1A | Mis | c.3994G>A, p.Ala1332Thr | LP | 4.3 | 1 | 16 | NL |
| Pt69 | 10.8 | M | SCN1A | Mis | c.580G>A, p.Asp194Asn | LP | - | 1 | 9 | NL |
| Pt70 | 13.6 | F | SCN1A | Fs | c.3800dup, p.Met1267IlefsTer27 | LP | - | 3 | ***12, 45, 81*** | HS (12m) |
| Pt71 | 10.8 | M | SCN1A | Spl | c.965-2A>G | LP | - | 2 | 6, ***14*** | CA (14m) |
| Pt72 | 10.4 | M | SCN1A | Spl | c.2589+2T>C | LP | - | 2 | ***6, 95*** | VE (6m) |
| Pt73 | 11.7 | F | SCN1A | Del | Exon 7-16 deletion | P | - | 1 | 33 | NL |
| Pt74 | 10.2 | M | SCN1A | Mis | c.4392G>C, p.Leu1464Phe | LP | 2.4 | 1 | 6 | NL |
| Pt75 | 12.3 | M | SCN1A | Mis | c.5297T>C, p.Val1766Ala | LP | 6.3 | 3 | 27, 50, 121 | NL |
| Pt76 | 22.2 | M | SCN1A | Non | c.644_655del, p.Leu215Ter | LP | - | 2 | 10, 167 | NL |
| Pt77 | 9.6 | F | SCN1A | Spl | c.4581+1G>T | LP | - | 1 | 3 | NL |
| Pt78 | 10.8 | M | SCN1A | Mis | c.580G>A, p.Asp194Asn | LP | 3.3 | 1 | 12 | NL |
| Pt79 | 9.3 | F | SCN8A | Mis | c.2549G>A, p.Arg850Gln | LP | 1.0 | 2 | 3, ***10*** | CA (10m) |
| Pt80 | 8.8 | M | SCN1A | Fs | c.727delT, p.Ser243LeufsTer2 | LP | 1.1 | 1 | 8 | NL |
| Pt81 | 9.1 | F | SCN1A | - | - | - | - | 5 | ***23, 24, 25, 26, 29*** | Progressive VE (from 23m) |
| Pt82 | 9.0 | M | SCN1A | Mis | c.1154A>G, p.Glu385Gly | LP | - | 3 | 4, ***6, 40*** | Progressive CA (from 6m) |
| Pt83 | 9.3 | F | SCN1A | - | - | - | - | 1 | 8 | NL |
| Pt84 | 9.1 | M | SCN1A | Non | c.2228G>A, p.Trp743Ter | P | 1.2 | 2 | ***14, 53*** | HS, DVA (from 14m) |
| Pt85 | 8.7 | F | SCN1A | Spl | c.2556+3A>T | LP | 2.8 | 2 | 5, ***76*** | CA (76m) |
| Pt86 | 8.3 | M | SCN2A | Mis | c.819C>A, p.Asn273Lys | LP | 1.1 | 1 | ***15*** | CA, CCA, VE (15m) |
| Pt87 | 9.3 | F | SCN1A | Mis | c.2552G>A, p.Arg851Gln | LP | 1.3 | 1 | 8 | NL |
| Pt88 | 8.4 | F | SCN2A | Mis | c.4622T>A, p.Ile1541Asn | LP | 0.5 | 2 | 2, 6 | NL |
| Pt89 | 8.3 | M | SCN1A | Mis | c.2804G>A, p.Arg935His | LP | 0.4 | 1 | ***5*** | Midbrain micro-hemorrahge |
| Pt90 | 8.7 | M | SCN1A | Mis | c.680T>G, p.Ile227Ser | LP | 1.0 | 2 | ***11, 69*** | Diffuse CA (from 11m) |
| Pt91 | 10.2 | F | SCN1A | Mis | - | - | - | 1 | 26 | NL |
| Pt92 | 9.8 | M | SCN1A | Del | c.4442_4443+1delAGA | LP | 2.0 | 1 | 22 | NL |
| Pt93 | 8.1 | M | SCN1A | - | - | - | - | 1 | 4 | NL |
| Pt94 | 8.8 | F | SCN1A | Mis | c.1033T>A, p.Cys345Ser | LP | 1.2 | 1 | 12 | NL |
| Pt95 | 7.8 | M | SCN2A | Mis | c.4886G>T, p.Arg1629Leu | LP | 0.2 | 4 | 0, ***1, 3, 13*** | HIE, PVL, CCA, VE (1m); Progressive WM-A & VE (13m) |
| Pt96 | 7.8 | M | SCN1A | Non | c.1738C>T, p.Arg580Ter | P | 0.9 | 1 | 8 | NL |
| Pt97 | 8.2 | M | SCN1A | - | - | - | - | 1 | 66 | NL |
| Pt98 | 9.6 | M | SCN2A | Fs | c.3171del, p.Thr1058ProfsTer2 | LP | 5.8 | 2 | ***26, 58*** | HAn - incomplete rotation (from 26m); CA, VE (58m) |
| Pt99 | 7.7 | M | SCN2A | Mis | c.4877G>T, p.Arg1626Leu | LP | 0.5 | 1 | ***6*** | Diffuse CA (6m) |
| Pt100 | 7.8 | F | SCN1A | Fs | c.3883delT, p.Tyr1295ThrfsTer13 | LP | 1.0 | 1 | 8 | NL |
| Pt101 | 8.1 | M | SCN1A | Mis | c.2804G>A, p.Arg935His | LP | 1.9 | 1 | 8 | NL |
| Pt102 | 8.3 | F | SCN1A | Mis | c.243C>A, p.Asp81Glu | LP | 1.1 | 1 | 5 | NL |
| Pt103 | 7.4 | M | SCN1A | Spl | c.4819+1G>A | LP | 0.7 | 1 | 6 | NL |
| Pt104 | 7.3 | M | SCN2A | Mis | c.5636T>C, p.Met1879Thr | LP | 0.2 | 1 | 1 | NL |
| Pt105 | 12.1 | M | SCN1A | Mis | c.4753C>T, p.Arg1585Cys | LP | 5.1 | 1 | 47 | NL |
| Pt106 | 8.3 | M | SCN3A | Spl | c.3393+2T>G | LP | 1.8 | 5 | 14, ***20, 23, 26, 47*** | Lt PL superficial siderosis (20m) |
| Pt107 | 7.4 | F | SCN8A | Mis | c.2934C>A, p.Ser978Arg | LP | 1.0 | 3 | 2, ***24, 52*** | Diffuse CA (24m); HS (52m) |
| Pt108 | 6.8 | F | SCN8A | Mis | c.2911C>G, p.Leu971Val | LP | 0.2 | 2 | 0, 2 | NL |
| Pt109 | 7.3 | M | SCN2A | Mis | c.658A>G, p.Arg220Gly | LP | 0.8 | 4 | 1, ***9, 11, 17*** | HIE, diffuse CA, VE (from 9m) |
| Pt110 | 7.0 | M | SCN2A | Mis | c.4426T>A, p.Phe1476Ile | LP | 0.4 | 1 | 3 | NL |
| Pt111 | 14.2 | F | SCN1A | Non | c.4834G>T, p.Glu1612Ter | LP | 7.8 | 2 | 102, ***144*** | Lt TenHyp, OL Hern (144m) |
| Pt112 | 6.7 | F | SCN2A | Mis | c.4499C>T, p.Ala1500Val | LP | 0.2 | 3 | 0, ***6, 18*** | FCD, CCA, diffuse WM-A, bilateral TA, VE (6m); progressive CA & VE (18m) |
| Pt113 | 6.8 | M | SCN1A | Non | c.3328G>T, p.Glu1110Ter | P | 1.4 | 2 | 7, 11 | NL |
| Pt114 | 10.3 | M | SCN1A | Del | exon 20-27 deletion | LP/LP | 4.3 | 2 | 9, 66 | NL |
| Pt115 | 6.3 | M | SCN2A | Mis | c.2990A>G, p.Asp997Gly | LP | 0.3 | 3 | ***0, 3,*** 36 | SDH |
| Pt116 | 6.8 | M | SCN1A | Del | exon 17-19 deletion | LP | 1.1 | 1 | 9 | NL |
| Pt117 | 6.3 | F | SCN1A | Spl | c.2382+1G>A | LP | 0.4 | 1 | 4 | NL |
| Pt118 | 11.3 | F | SCN1A | - | - | - | - | 2 | ***30, 66*** | HIE, diffuse CA (30m); progressive CA, Cb-A, VE (66m) |
| Pt119 | 6.0 | M | SCN1A | Mis | c.4460T>C, p.Ile1487Thr | LP | 0.2 | 4 | ***1, 3, 9, 15*** | SDH (1m); VE (3m); CA (9m) |
| Pt120 | 6.3 | F | SCN1A | Mis | c.4822G>T, p.Asp1608Tyr | LP | - | 1 | ***12*** | Prominent Rt CCV (12m) |
| Pt121 | 6.8 | F | SCN1A | Non | c.5734C>T, p.Arg1912Ter | P | - | 1 | 13 | NL |
| Pt122 | 6.3 | M | SCN1A | Mis | c.530G>T, p.Gly177Val | LP | 0.7 | 1 | 6 | NL |
| Pt123 | 9.2 | F | SCN1A | Mis | c.4264G>C, p.Gly1422Arg | LP | 3.7 | 1 | 44 | NL |
| Pt124 | 18.7 | F | SCN1A | - | - | - | - | 2 | ***89, 160*** | HS (from 89m) |
| Pt125 | 5.5 | F | SCN8A | Mis | c.4398C>A, p.Asn1466Lys | LP | 0.2 | 1 | ***1*** | WM-SA (1m) |
| Pt126 | 20.6 | F | SCN2A | Spl | c.3400-2A>G | LP | 15.4 | 3 | 42, 57, ***180*** | FCD (180m) |
| Pt127 | 5.7 | M | SCN2A | Mis | c.2558G>A, p.Arg853Gln | LP | 0.5 | 2 | ***7, 20*** | CCA, WM-A, WM-SA (7m); CA (20m) |
| Pt128 | 5.7 | F | SCN1A | Mis | c.841C>T, p.Pro281Ser | LP | - | 1 | 12 | NL |
| Pt129 | 5.0 | F | SCN1A | Spl | c.602+1G>C | P | - | 1 | 2 | NL |
| Pt130 | 5.3 | M | SCN1A | Spl | c.602+1G>A | P | - | 1 | 9 | NL |
| Pt131 | 4.4 | F | SCN8A | Mis | c.2934C>A, p.Ser978Arg | LP | 0.3 | 1 | 1 | NL |
| Pt132 | 4.8 | M | SCN1A | - | - | - | - | 1 | 8 | NL |
| Pt133 | 4.2 | M | SCN2A | Mis | c.3956G>A, p.Arg1319Gln | LP | 0.3 | 1 | 4 | NL |
| Pt134 | 5.8 | F | SCN1A | - | - | - | - | 1 | ***17*** | WM-SA (17m) |
| Pt135 | 14.1 | F | SCN1A | Spl | c.2947-1G>A | P | - | 1 | 59 | NL |
| Pt136 | 3.9 | M | SCN2A | Mis | c.3631G>A, p.Glu1211Lys | LP | 0.5 | 1 | 6 | NL |
| Pt137 | 4.2 | F | SCN8A | Mis | c.4391T>C, p.Ile1464Thr | LP | 0.8 | 1 | 8 | NL |
| Pt138 | 4.3 | M | SCN1A | - | - | - | - | 1 | 13 | NL |
| Pt139 | 4.2 | M | SCN1A | - | - | - | - | 1 | 11 | NL |

Abbreviations

d, day(s); m, month(s); y, year(s); &, and; -, not applicable or available.

F, female; M, male; NL, normal; Lt, left; Rt, right.

Del, deletion; Fs, frameshift; Ins, insertion; LP, likely pathogenic; Mis, missense; Non, nonsense; P, pathogenic; Spl, splice site.

AC, arachnoid cyst; AmyE, amygdala enlargement; CA, cerebral atrophy; Calcn, calcification; Cb-A, cerebellar atrophy; Cb-SA, cerebellar signal abnormalities; CCA, corpus callosum atrophy/thining; CCV, cerebral cortical veins; FCD, focal cortical dysplasia; FL, frontal lobe; HAn, hippocampal anomly; Hern, hernation; HIE, hypoxic-ischemic encephalopathy or hypoxic-ischemic encephalopathy-like; HS, hippocampal sclerosis; Mb, midbrain; mHemo, micro-hemorrhage; OL, occipital lobe; PL, parietal lobe; PV, periventricular; PVL , periventricular leukomalacia; SDH, subdural hemorrhage; TA, thalamal atrophy; TenHyp, tentorial hypoplasia; VE, ventriculomegaly; WM-A, white matter atrophy; WM-SA, white matter signal abnormalities.

*Ages with abnormal MRI findings are denoted by bold, italicized, and underlined text.
